# Non-Markov-Type Analysis and Diffusion Map Analysis for Molecular Dynamics Trajectory of Chignolin at a High Temperature

**DOI:** 10.3390/life12081188

**Published:** 2022-08-03

**Authors:** Hiroshi Fujisaki, Hiromichi Suetani, Luca Maragliano, Ayori Mitsutake

**Affiliations:** 1 Department of Physics, Nippon Medical School, 1-7-1 Kyonan-cho, Musashino, Tokyo 180-0023, Japan; 2 AMED-CREST, Japan Agency for Medical Research and Development, 1-1-5 Sendagi, Bunkyo-ku, Tokyo 113-8603, Japan; 3 Department of Integrated Science and Technology, Faculty of Science and Technology, Oita University, 700 Dannoharu, Oita 870-1192, Japan; suetani@oita-u.ac.jp; 4 Department of Life and Environmental Sciences, Polytechnic University of Marche, 60131 Ancona, Italy; l.maragliano@staff.univpm.it; 5 Center for Synaptic Neuroscience and Technology, Italian Institute of Technology, 16132 Genova, Italy; 6 Department of Physics, School of Science and Technology, Meiji University, 1-1-1 Higashi-Mita, Tama-ku, Kawasaki 214-8571, Japan; ayori@meiji.ac.jp

**Keywords:** molecular dynamics simulation, rare event, Markov state model, non-Markov-type analysis, diffusion map, weighted ensemble simulation

## Abstract

We apply the non-Markov-type analysis of state-to-state transitions to nearly microsecond molecular dynamics (MD) simulation data at a folding temperature of a small artificial protein, chignolin, and we found that the time scales obtained are consistent with our previous result using the weighted ensemble simulations, which is a general path-sampling method to extract the kinetic properties of molecules. Previously, we also applied diffusion map (DM) analysis, which is one of a manifold of learning techniques, to the same trajectory of chignolin in order to cluster the conformational states and found that DM and relaxation mode analysis give similar results for the eigenvectors. In this paper, we divide the same trajectory into shorter pieces and further apply DM to such short-length trajectories to investigate how the obtained eigenvectors are useful to characterize the conformational change of chignolin.

## 1. Introduction

The kinetic description of (bio)molecules is inevitable to understand their chemical reactions or conformational change, but it is still difficult to thoroughly understand such transition processes due to the limitations of experimental and computational means. Recently, in particular for numerical simulations of biomolecules, the Markov state model (MSM) [1] has been often employed to analyze the kinetic properties of molecules, such as reaction rates and reaction pathways. A good thing about the MSM is its conceptual simplicity and ease of application. By calculating the so-called “transition matrix” (as described below), we can estimate the rate as an inverse of a mean first passage time (MFPT) between two states of concern. Furthermore, using transition path theory [2], we can also estimate dominant pathways using a committor function [3,4]. However, there are at least three issues in the MSM, which are (1) the “lag time” (this represents a time interval between observations of some quantities in a trajectory) dependence of the result, (2) the state definition dependence of the result, and (3) the effects of the finiteness of the trajectory data. These are why many researchers have been developing new or improved methods for calculating reaction rates and other kinetic properties.

One such method is non-Markov-type analysis recently introduced by Zuckerman and coworkers [5,6]. This method is an extension of the conventional MSM, which lifts Problems (1) and (2) above, and the rate estimations can be robust, as shown in [5,6]. However, this type of analysis needs a very long-time trajectory or bunches of trajectories to robustly estimate kinetic properties. To overcome this issue, transition path sampling (TPS) [4] is often employed; however, the original idea of TPS is too demanding, and we need to employ “easier” path sampling methods based on collective variables (CVs), such as partial path sampling [7], forward flux sampling [8], or the weighted ensemble (WE) method [9]. We have applied the WE method to several proteins including chignolin [10,11,12] and estimated the rate constants between two metastable states. Hence, it is interesting to compare the rate constants using different computational methods, which is one of the concerns in this paper.

Another concern is how to choose “optimal” CVs. For biomolecules, CVs are often chosen based on chemical intuitions or traditional ideas, but recently, machine learning or manifold learning techniques have become popular to extract CVs. Historically, principal component analysis (PCA) has been used over the years, but there are several problems in PCA, so many researchers have been devising more advanced approaches such as relaxation mode analysis (RMA) [13,14,15,16,17], time-structured independent component analysis (tICA) [18,19,20], the isomap [21,22,23], the diffusion map (DM) [24,25,26], and many others. It is assumed that the kinetic properties are not so sensitive to the choice of the CVs (as exemplified in the reaction flux formalism [4,27]), but “optimal” CVs should be better for both the convergence of calculations and the interpretations of the results. Previously, we used DM for a long-time trajectory of chignolin at a high temperature (420 K) [10] and found that (1) the first few DM eigenvectors well correlated with eigenvectors calculated from RMA, (2) the second DM eigenvector correlated most with the dihedral angle of glycine in chignolin, and (3) the efficiency to calculate the kinetic properties of chignolin does not seem to depend on whether we choose hydrogen bond distances or DMs as CVs. The trajectory analyzed was long enough (∼750 ns) to sample the whole conformational space at the folding temperature, but it is not always the case when we attack bigger or longer-time scale problems. Hence, it is always important to consider what we can learn from shorter trajectories about the global information of the conformational space. Dividing the same trajectory into shorter pieces, we here investigate the kinetic properties of chignolin from shorter-time perspectives, hoping to connect with the enhanced sampling techniques such as the weighted ensemble method [9].

This paper is organized as follows. In Section 2, we briefly describe the methodologies (non-Markov-type analysis and diffusion map analysis) used here for the investigation of the kinetic properties of a small protein (chignolin). In Section 3, after describing the simulated system, we present numerical results for the kinetic properties of chignolin and discuss the connection with the previous results. In Section 4, we conclude the paper.

## 2. Methods

### 2.1. Non-Markov-Type Analysis

Recently, Zuckerman and coworkers advocated for a new trajectory analysis method called non-Markov-type analysis [5,6], which is an extension of the conventional MSM [1]. We will briefly summarize it here for completeness.

The basic quantity for the Markov-type analysis is the transition matrix $T_{ij}$ calculated as [1]:

(1) $$\begin{array}{c} T_{ij}=\frac{N_{ij}}{\sum_{j}N_{ij}} \end{array}$$

where $N_{ij}$ is the counting matrix between states *i* and *j*, which is directly enumerated from a given trajectory with a given lag time τ (hereafter, we omit the τ dependence for variables such as $N_{ij}$ and $T_{ij}$). It is well known that from this transition matrix, we can calculate the equilibrium population $P_{i}^{\mathrm{eq}}$ for each state *i* as:

(2) $$\begin{array}{c} P_{i}^{\mathrm{eq}}=\sum_{j}P_{j}^{\mathrm{eq}}T_{ji} \end{array}$$

and the mean first passage time (MFPT) $F_{if}$ from state *i* to *f* as

(3) $$\begin{array}{c} F_{if}=1+\sum_{j\neq f}T_{ij}F_{jf}, \end{array}$$

where time is measured in units of the lag time τ. These two relations are most relevant for the practical application of the MSM. The latter relation is proven as follows. We can define the first passage time distribution from state *i* to *j* over *n* steps $f_{ij}^{\left(n\right)}$ as

(4) $$\begin{array}{c} f_{ij}^{\left(n\right)}=\sum_{k\neq j}T_{ik}f_{kj}^{\left(n-1\right)}. \end{array}$$

where $f_{ij}^{\left(n\right)}$ is recursively defined using the following relations:

(5) $$\begin{array}{c} f_{ij}^{\left(1\right)}=T_{ij},\;f_{ij}^{\left(2\right)}=\sum_{k\neq j}T_{ik}f_{kj}^{\left(1\right)},\;\cdots . \end{array}$$

From these distributions, the MFPT is defined as

(6) $$\begin{array}{c} F_{ij}=\sum_{n=1}^{\infty }nf_{ij}^{\left(n\right)} \end{array}$$

and just the rearrangement of the terms leads to Equation (3).

In the non-Markov-type analysis [5,6], we keep track of which state a trajectory is in until it transits to other states, so there remains a kind of memory in the analysis (this is why we call it non-Markov). For concreteness, we take a three-state model (A, I, B) and construct the transition matrix from state A to B, $T^{A\to B}$, as

(7) $$\begin{array}{c} T^{A\to B}=\left(\begin{array}{ccc} T_{11}^{\mathrm{AA}} & T_{12}^{\mathrm{AA}} & T_{13}^{\mathrm{AB}}\\ T_{21}^{\mathrm{AA}} & T_{22}^{\mathrm{AA}} & T_{23}^{\mathrm{AB}}\\ 0 & 0 & 1 \end{array}\right). \end{array}$$

Here, $T_{ij}^{\mu \nu }$ is a *conditional* transition matrix where the last state is μ(=A) and the next entering state is ν(=A,B), and i,j runs through (1,2,3), which are identified as (A,I,B). From this transition matrix, we can calculate the first passage time distribution and MFPT as in the case of the conventional MSM.

There is a similar method called core-set MSM [28], which is an extension of the milestoning method [29] using the idea of a “core set”. We found that the results obtained are similar for the system analyzed, so we here only show the numerical results using the non-Markov-type analysis.

### 2.2. Diffusion Map Analysis

The diffusion map (DM) is a manifold-learning method, which was invented by Coifman and coworkers [24] and since then has been applied to many problems including image classification, speaker classification, and so on. The basic idea is to extract a low-dimensional manifold embedded in a high-dimensional data space, and to this end, we construct a matrix, which will be diagonalized. The construction goes as follows. Given we have time series data or a data ensemble, where the dimension of the data space is *M* and the number of samples is *N*, i.e., we have $x_{i}\in R^{M}\left(i=1,\cdots ,N\right)$, we firstly consider the following Gaussian kernel:

(8) $$\begin{array}{c} k\left(x_{i},x_{j}\right)=\exp\left(-\frac{\left|\right|x_{i}-x_{j}{\left|\right|}^{2}}{2\epsilon }\right) \end{array}$$

where ||·|| is a metric and a normal Cartesian metric is usually employed. ϵ is a hyperparameter, which is tuned by some criteria. From this kernel, we next construct the N×N “transition matrix” $M_{ij}$ as follows:

(9) $$\begin{array}{c} M_{ij}=\frac{k(x_{i},x_{j})}{p(x_{i})} \end{array}$$

with

(10) $$\begin{array}{c} p\left(x_{i}\right)=\sum_{j}k\left(x_{i},x_{j}\right). \end{array}$$

This form looks like a transition matrix $T_{ij}$ in the MSM defined above because both $N_{ij}$ and $k(x_{i},x_{j})$ represent a propensity to make a move from state *i* to *j*. Another construction starts from defining the following matrix:

(11) $$\begin{array}{c} K_{ij}=\frac{k(x_{i},x_{j})}{\sqrt{p\left(x_{i}\right)p\left(x_{j}\right)}}, \end{array}$$

and in this case, a transition matrix $M_{ij}$ is defined as

(12) $$\begin{array}{c} M_{ij}^{*}=\frac{K_{ij}}{\sum_{j}K_{ij}}. \end{array}$$

It is known that regarding this form as a propagator for a density function, the backward Fokker–Planck (FP) equation is obtained in the N→∞,ϵ→0 limit. However, notice that the time series data analyzed do not necessarily have such a stochastic character that the data are generated by the backward FP equation. Since the eigenvalues and eigenvectors calculated from $M_{ij}$ and $M_{ij}^{*}$ are qualitatively similar, we use the first transition matrix $M_{ij}$ (9) for the numerical analysis of the trajectory data.

By diagonalizing $M_{ij}$ with $\sum_{j}M_{ij}u_{\alpha }\left(x_{j}\right)=\lambda_{\alpha }u_{\alpha }\left(x_{i}\right)$, we obtain the eigenfunctions $u_{\alpha }\left(x_{i}\right)$ and eigenvalues $\lambda_{\alpha }$. There is the following property that $\lambda_{1}=1>\lambda_{2}>\lambda_{3}>\cdots$, and $u_{1}\left(x\right)$ represents the equilibrium distribution as in the case of the MSM. As the CVs in this paper, we decided to take the second and third DM coordinates ($\lambda_{2}^{t}u_{2}\left(x\right),\lambda_{3}^{t}u_{3}\left(x\right))$ where *t* is time measured in units of the lag time (for simplicity, we take t=1 in this paper).

## 3. Results and Discussion

### 3.1. On Chignolin and Simulation Setup

**Figure 1:**
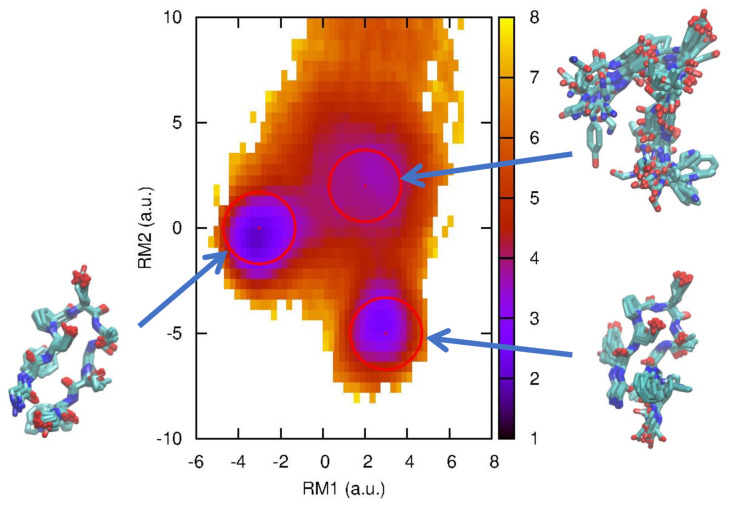
Free energy landscape of chignolin at 420 K along the first and second relaxation mode (RM) coordinates. The folded (native), misfolded, and intermediate states are indicated by circles with a radius of 1.4 (a.u.). The multiple typical structures corresponding to these states are also depicted using VMD [39].

The molecular system we used here is a small peptide, chignolin (PDBID: 1UAO), which is an artificially synthesized peptide [30] with only 10 amino acids (GYDPETGTWG). It is known that this is one of the smallest peptides that has a unique fold (native state) [30], so it can be regarded as a “mini-protein”. After its discovery, chignolin has been studied by many researchers with MD simulations and has been used to examine new simulation algorithms and protocols. The free energy landscape using two hydrogen bond (HB) distances was calculated by Terada and coworkers using the multicanonical sampling method [31] and multiscale enhanced sampling method [32], and it was found that there is a misfolded state where the HB configuration is different from that in the native state (Figure 1). These native and misfolded states were obtained by other researchers [33,34,35].

Note that there are two types of chignolin, the above “original” chignolin [30] and a mutated one called CLN025 (PDBID: 2RVD, 5AWL) with amino acid sequence YYDOPETGTWY [36]. The dynamics of CLN025, as well as other small peptides and proteins was extensively studied by D.E. Shaw’s research group using their Anton hardware [37]. Zuckerman’s group used the Anton data to clarify the folding mechanism and folding rate of CLN025 and other peptides at room temperature [5]. The MD simulations of CLN025 showed that CLN025 has a simple two-state folding (native and unfolding) mechanism without a misfolded state. Here, we will examine the “original” chignolin, which has somewhat complicated folding pathways.

Directly related to our study, one of the authors (A.M.) performed an MD simulation of the original chignolin near its folding transition temperature and showed the effectiveness of relaxation mode analysis (RMA) [38], which extracts slow relaxation modes and their associated timescales from simulation data. Historically, RMA was developed to examine the “dynamic” properties of spin systems [13] and homopolymer systems [14,15], but has also been recently applied to biomolecular systems [16,17,38]. (RMA is similar to time-structure-based independent component analysis (tICA) in [18,19,20], but tICA is a special case of RMA with $t_{0}$ = 0. From RMA, the concept of slow relaxation is naturally introduced. See the conclusion of [16] for more details about the difference between tICA and RMA.) In [38], the free energy landscape using slow modes obtained by RMA was calculated and an intermediate state was found in addition to the previously found native and misfolded states, as shown in Figure 1. Here, we use the same trajectory data of chignolin in [38], so the setup of the molecular dynamics calculation is the same as well [38]. An MD simulation, augmented by a GPGPU, was performed using the AMBER package (AMBER 11.0) with the ff99SB force field and TIP3P water model. An extended structure was solvated with a 15 Å buffer of TIP3P water around the peptide in each dimensional direction. The numbers of atoms of the peptide and water molecules are 138 and 10,941 (3647 water molecules), respectively. Two potassium ions (Na${}^{+}$) are included in the system, resulting in a net-neutral system. The total number of atoms in the system is 11,081, and a 750 ns MD production run at 1 atm pressure and a 420 K temperature (near folding temperature) was performed with a time step of 2 fs. The Langevin thermostat with a friction constant γ=2.0 ps${}^{-1}$ was used for temperature control. For analysis, the coordinates were saved every 10 ps, and the total number of samples was 75,000. The free energy landscape of chignolin along the first and second slowest relaxation mode (RM) directions is shown in Figure 1.

### 3.2. First Passage Time Distributions and Transition Rates

We here evaluate the first passage time distributions (FPTDs) using the non-Markov-type analysis introduced above. From the free energy landscape in Figure 1, we define three, folded (F), misfolded (M), and intermediate (I), states whose centers are (−3.0, 0.0), (3.0, −5.0), and (2.0, 2.0), respectively with a radius of 1.4 (the rest is regarded as an unfolded state). We then count the transitions between these states and construct the conditional transition matrix $T_{ij}^{\mu \nu }$ with a lag time of 10 ps. Using Equations (3) and (4), we can calculate the first passage time distribution and MFPT, respectively. For comparison, we also employ a “naive” method to calculate the first passage time distribution as follows: We pick $x_{i}$, which is classified as state A, and then, search along the time series when it makes a first transition to state B. When it happens at $x_{j}$, we then calculate the FPT from state A to B as (j−i)τ where τ is the lag time.

**Figure 2:**
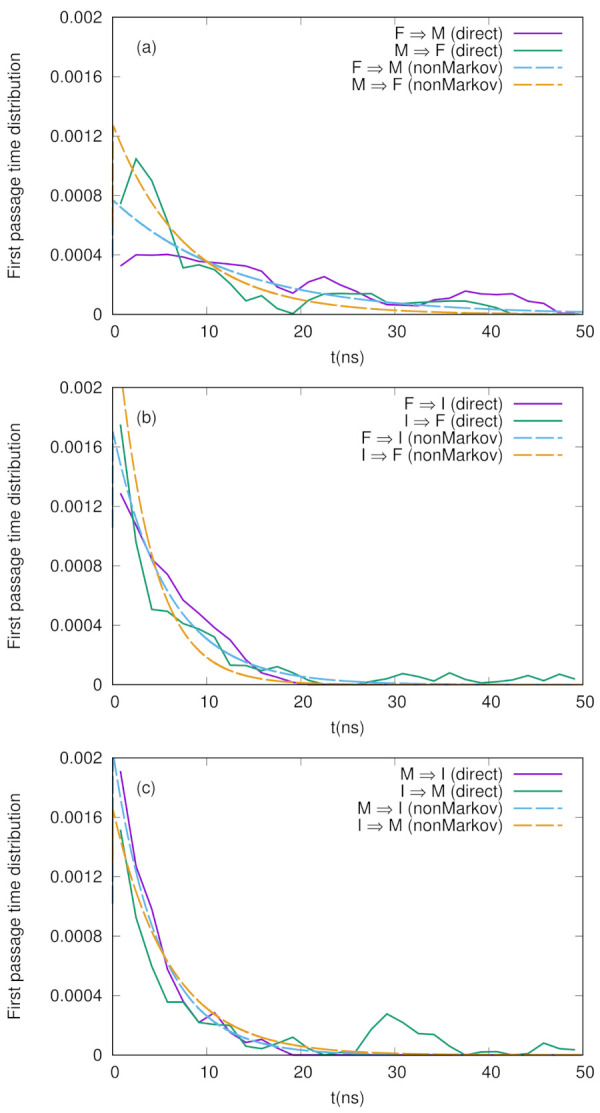
First passage time distributions between three states (F, M, and I) defined in Figure 1 calculated by non-Markov-type analysis and the naive method explained in the text. (**a**) F ↔ M, (**b**) F ↔ I, and (**c**) M ↔ I. The MFPTs calculated by the non-Markov analysis are $F_{\mathrm{FM}}=13$ ns, $F_{\mathrm{MF}}=7.8$ ns, $F_{\mathrm{FI}}=5.9$ ns, $F_{\mathrm{IF}}=3.7$ ns, $F_{\mathrm{MI}}=4.9$ ns, and $F_{\mathrm{IM}}=6.0$ ns.

**Figure 3:**
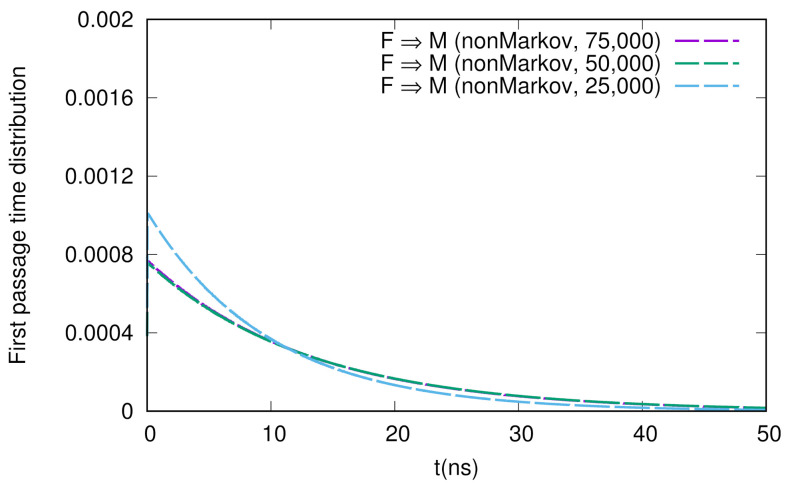
Sample number dependence of the first passage time distribution for the F → M transition. The numbers of samples used here are 75,000, 50,000, and 25,000.

We show the numerical results for FPTDs among *F*, *M*, and *I* in Figure 2. Basically, the order of the time scales are ∼10 ns (for detail, see the caption in Figure 2), and there are slight differences between the forward and backward transitions. We also notice that the naive method agrees well with the non-Markov-type analysis, though there are large fluctuations in the naive method. We believe that we need much longer simulations to obtain fully converged results when we use the naive method. On the other hand, when we use the non-Markov analysis, the convergence seems to be faster (shorter simulations give a reasonable result), as shown in Figure 3.

We previously estimated the MFPTs for the conformational change dynamics of chignolin using the weighted ensemble (WE) method [10], and it turned out that the time scales for MFPTs obtained were also ∼10 ns when we assumed a linear regression for the population dynamics. (In the previous paper, we obtained shorter time scales for relaxation using a three-state kinetic model, but such time scales are not directly related to the MFPTs.) Hence, we conclude that the previous WE simulation is consistent with the present analysis.

### 3.3. Correlations between Dihedral Angles of Chignolin and Collective Variables

**Figure 4:**
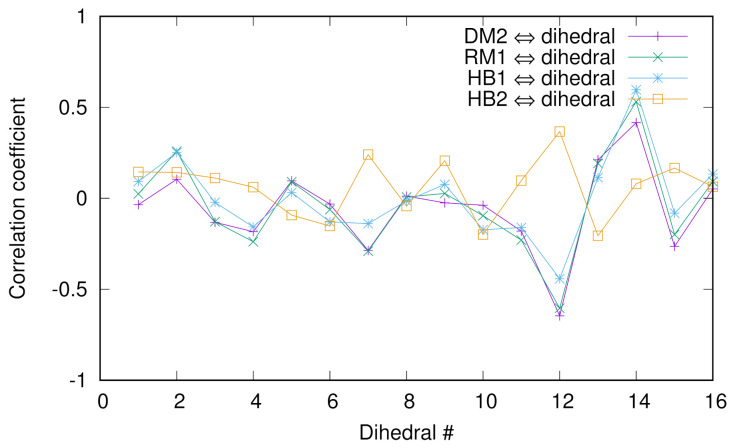
The Pearson correlation coefficients between several collective variables (second DM coordinate, first RM coordinate, and two hydrogen bond distances between Asp3O and Gly7N named HB1 or between Asp3N and Thr8O named HB2) and 16 dihedral angles (ϕ,ψ) of chignolin.

Previously, we employed the diffusion map (DM) method to extract the collective variables (CVs) of chignolin [10] and discussed the correlation between a dihedral angle of glycine and the DM coordinates and relaxation mode (RM) coordinates. To look into more detail of such correlations, we calculated the Pearson correlation coefficients between several collective variables (second DM coordinate, first RM coordinate, and two hydrogen bond distances between Asp3O and Gly7N named HB1 or between Asp3N and Thr8O named HB2) and 16 dihedral angles (ϕ,ψ) of chignolin in Figure 4. For the numerical protocols for the DM and RM coordinates, see the previous papers [10,38]. We see that except HB2, the correlations are good, indicating that HB1 is a “good” CV since we know that the second DM and first RM coordinates are good CVs. In addition, the absolute values of the coefficients are the largest at the 12th and 14th angles (except HB2), which are the ψ’s of glycine and threonine, indicating the importance of these two residues for conformational change.

The cross-correlations between the DM and RM coordinates are shown below. We see that the correspondence between DM2 and RM1 or DM3 and RM2 is good, but that between DM4 and RM3 is less significant.

$$\begin{array}{cc} & \begin{array}{ccc} \mathrm{DM}2 & \mathrm{DM}3 & \mathrm{DM}4 \end{array}\\ \begin{array}{c} \mathrm{RM}1\\ \mathrm{RM}2\\ \mathrm{RM}3 \end{array} & \left(\begin{array}{ccc} 0.87 & -0.25 & 0.12\\ -0.19 & -0.81 & 0.09\\ 0.13 & -0.03 & -0.57 \end{array}\right) \end{array}$$

### 3.4. Short-Time Diffusion Map Analysis for Chignolin

Finally, we show a different type of analysis using the DM method, that is short-time diffusion map analysis. Clementi et al. [25] and Trstanova et al. [26] have used this type of analysis for different molecular systems, and we here apply this method to chignolin dynamics. The basic idea is simple and trivial: we chop a long-time trajectory into shorter pieces, and apply the DM method to each short piece of the trajectories. As shown in [26], DM coordinates extracted by such short-time DM can approximate the local equilibrium dynamics of the system, and furthermore (more importantly), as shown in [25], the short-time DM might be able to extract the directions of conformational change, which can be further used for sampling. This kind of idea was recently elucidated by Morishita [40], but his idea is to combine short-time principal component analysis [41] with sampling. Here, we examine chignolin dynamics in terms of short-time DM analysis.

**Figure 5:**
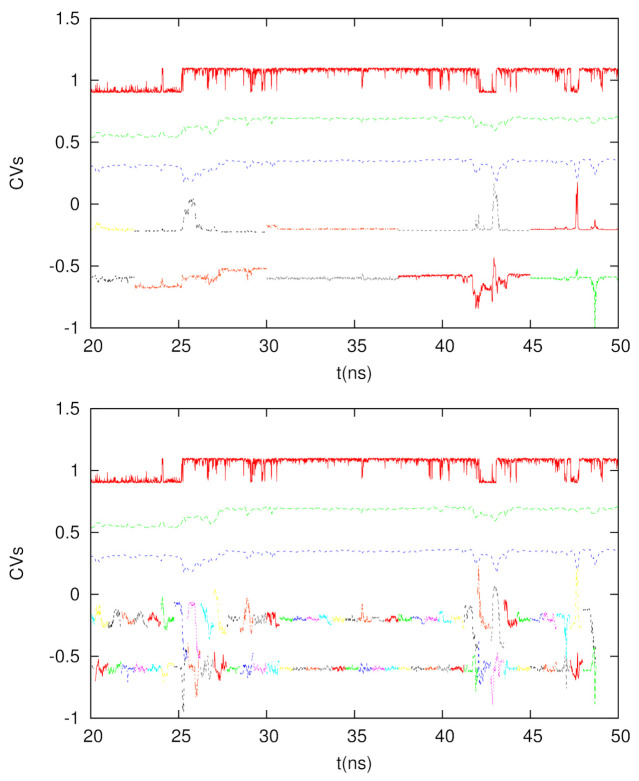
From top to bottom for each panel: The dihedral angle of glycine in chignolin (red), 2nd DM coordinate from the full trajectory (green), 3rd DM coordinate from the full trajectory (blue), 2nd DM coordinate from the shorter trajectories (multiple colors for different trajectory segments), and 3rd DM coordinate from the shorter trajectories (multiple colors for different trajectory segments). Top panel: The time interval is 7.5 ns. Bottom panel: The time interval is 0.75 ns.

We tested two time intervals to calculate the DM coordinates, which are 7.5 ns and 0.75 ns. Since 10 ps is the time interval to save the trajectory for chignolin, each DM matrix is 750 × 750 and 75 × 75, respectively. In Figure 5, we depict the time courses of the second and third short-time DM coordinates, as well as the conventional DM coordinates (calculated by the full trajectory, but with a time interval of 100 ps) and the glycine dihedral angle. We see if we use a longer time interval (7.5 ns), the behavior of the short-time DM is similar to that of the long-time DM, though the correlations between short-time and long-time DM can interchange between DM2 and DM3 (for example, the time duration between 25 and 30 ns). If we use a shorter time interval (0.75 ns), such a correspondence is less significant, but we can see that at the transitions, the fluctuations of the DM2 and DM3 coordinates become large, indicating the usefulness of the short-time DM to detect rare events. Hence, if we use the short-time DM to extract the tentative CVs for conformational change or rare events, it will be possible to enhance the sampling of the conformational space or the calculations of the kinetic properties using these coordinates.

## 4. Concluding Remarks

We analyzed a 750 ns-long molecular dynamics trajectory of chignolin, a small peptide with 10 amino acids, in terms of kinetic properties. There are three particular metastable states in chignolin, folded, misfolded, and intermediate states, and the first passage time distributions between these states were estimated using the non-Markov-type analysis and the naive method. The estimated mean first passage times are ∼10 ns, which is comparable to the time scales calculated by the weighted ensemble method. We also applied the short-time diffusion map analysis to the same trajectory and found that the DM coordinates calculated from short-time trajectories correlate well with those calculated from a long-time trajectory, and even if we use a short-time interval (0.75 ns), the conformational change or rare events can be detected as the large fluctuations of the DM coordinates.

We mention further issues related to this study: We here used the previous trajectory at a high temperature to accelerate the convergence and computation, but if the temperature decreases, the computation becomes harder because of the existence of high free energy barriers. For lower temperatures or bigger systems, we should use more powerful computational resources such as Anton [37] or accelerated simulation methods such as the weighted ensemble method [9], keeping the kinetic properties of the system intact. One idea is to use short-time DM analysis to extract good CVs, which are further combined with the weighted ensemble method for more efficient sampling of the kinetics.

## Data Availability

Not applicable.

## Abbreviations

The following abbreviations are used in this manuscript:

MD	Molecular dynamics
CV	Collective variable
DM	Diffusion map
WE	Weighted ensemble
RM	Relaxation mode
